# The use of compression stockings for venous disorders in Brazil

**DOI:** 10.1258/phleb.2011.010088

**Published:** 2012-02

**Authors:** J L Cataldo, J M Pereira de Godoy, N de Barros

**Affiliations:** * Service of Peripheral Vascular Disease, University of Campinas UNICAMP; † Department of Cardiology and Cardiovascular Surgery, Medicine School of São Jose do Rio Preto-FAMERP, Brazil; ‡ Discipline of Vascular Surgery, Federal University of São Paulo-UNIFESP, Brazil

**Keywords:** compression therapy, chronic venous diseases, elastic stockings

## Abstract

**Objective:**

The aim of this study was to analyse the medical indication and the use of elastic compression stockings, and to assess patient adherence to treatment in different regions of Brazil.

**Method:**

The prescription and clinical indication of elastic stockings were evaluated in a prospective, descriptive, cross-sectional, multicentre study for a population of private patients. In 2009, 3414 patients from 123 treatment centres in southern, south-eastern and north-eastern Brazil were evaluated using a questionnaire. The following variables were analysed: sociodemographic (gender, age, occupation and education), lifestyle (physical activity and time spent standing); classification of venous disease (CEAP [clinical, aetiological, anatomical and pathophysiological] classification – clinical criteria), indications for prescription, consumer behaviour (strength, acquisition and use of stockings) and criteria of satisfaction (improvement, duration of use, adherence). The effects of compression therapy were assessed at a follow-up visit approximately 30 days after starting treatment with the following items being assessed: complaints about pain, discomfort, burning sensation and oedema of the leg while using elastic stockings. Multivariate analysis was used to compare data with an alpha error of 5% (*P* value < 0.05) being considered acceptable.

**Results:**

The average age increased with the severity of chronic venous insufficiency; the main indications used by physicians were leg pain and discomfort; 89.3% of patients bought stockings and thus started treatment with more than 90% of these reporting improvements in symptoms.

**Conclusion:**

Elastic stockings are available to the Brazilian population, look acceptable at the time of purchase and provide good results; however, some limitations regarding their use need to be addressed.

## Introduction

The clinical signs of chronic venous disorders (CVDs) of the lower extremities range from small varicose veins to venous ulcers. Other signs include oedema, venous eczema, hyperpigmentation, white atrophy and lipodermatosclerosis with ulceration.^1–3^ Due to this diversity of clinical presentations, venous disorders are categorized by the clinical, aetiological, anatomical and pathophysiological (CEAP) classification system.^4^ This classification comprises six degrees of severity with grade I being the least severe form of the disease (telangiectasias and reticular veins) and grade VI being the presence of active venous ulcers.

Venous disease may affect 20–30% of the population with the least severe varicose veins possibly affecting around 50% of the population in Brazil.^5,6^

Compression therapy using elastic stockings is currently accepted as the most effective form of treatment to prevent or to delay the deterioration of skin and subcutaneous lesions that occur in the natural history of CVD.^6^ There is varying degrees of evidence (categories A and B) that demonstrate the real value of elastic compression in the treatment of CVDs.^2,3,7^

The true situation of the use of elastic compression stockings in everyday clinical practice is not known in Brazil nor is the level of adherence to treatment by patients with venous diseases. These data may be useful for specialists when prescribing treatment. The aim of this study was to analyse the medical indications and use of elastic compression stockings and to assess patient adherence to treatment.

## Method

The prescription, clinical indication and evolution of the use of elastic stockings for a population of private patients were evaluated in a prospective, descriptive, cross-sectional, multicentre study. In 2009, 3414 patients from 123 treatment centres in southern, south-eastern and north-eastern Brazil were evaluated using a questionnaire.

The centres were invited to participate after the objectives of the research were outlined. No fees were offered to the centres or to their patients. All patients who had symptoms and clinical diagnosis of chronic venous insufficiency (CVI) and were in conditions to use elastic compression were invited to participate in the study by order of arrival at the clinics. Patients with chronic arterial insufficiency and those who were unwilling to use the compression garment for any reason, for example, due to prior history of intolerance, were excluded. All patients were instructed to purchase and use one elastic stocking manufactured in Brazil by *Sigvaris* in order to standardize the price and pattern. The compression classes and size of the stockings employed in each treatment centre depended on the diagnosis and indication as suggested in the literature for each disease.

The following variables were analysed: sociodemographic (gender, age, occupation and education), lifestyle (physical activity and time spent standing); classification of venous disease (CEAP classification – clinical criteria); indications for prescription; consumer behaviour (strength, acquisition and use of stockings); and satisfaction criteria (improvement, duration of use, adherence). A physical examination using the CEAP criteria was employed in the diagnosis of CVI. The effects of compression therapy were assessed at a follow-up visit approximately 30 days after starting treatment with the following items being assessed: complaints about pain, discomfort, burning sensation and oedema of the leg while using elastic stockings. Oedema was diagnosed clinically and the other data were collected by interviewing the patients and from the medical history.

Multivariate analysis was used to compare data in the statistical analysis with an alpha error of 5% (*P* value < 0.05) being considered acceptable.

The study was approved by the Research Ethics Committee of UNIFESP – EPM (#1067-09 dated 14 August 2009).

## Results

A total of 3414 patients (80.5% women and 19.5% men) were included in the study with mean ages of 45.6 years for men and 47.4 years for women.

A total of 87.4% of the participants worked and 12.6% were retired; 24% had only completed junior high school, 44.3% had completed senior high school and 31.7% had college degrees.

Multivariate analysis demonstrated that there is no association between the severity of CVI according to the CEAP classification and gender, except for CEAP C1 where it was found that 15% of women and 8% of men had only telangiectasias and reticular veins.

Most of the patients with clinical indication for stockings had either CEAP C2 or C3, as is illustrated by Table 1.

**Table 1:** Sample distribution according to the CEAP clinical criteria

CEAP classification	*N*	%	95% confidence interval
C0	126	3.7	3.1–4.4
C1	451	13.2	12.0–14.3
C2	1393	40.8	39.1–42.2
C3	857	25.1	23.7–26.6
C4	451	13.2	12.0–14.3
C5	102	3.0	2.5–3.6
C6	34	1.0	0.7–1.3
Total	3414	100.0	

CEAP, clinical, aetiological, anatomical and pathophysiological

On comparing clinical severity according to the CEAP classification and age, multivariate analysis demonstrated that the severity of CVI increased as the age increased (Table 2).

**Table 2:** CEAP classification versus age

CEAP	Mean age (years)	Standard deviation (years)	Minimum (years)	Maximum (years)
C0	39.5	14.7	14	80
C1	40.5	14.3	15	85
C2	44.2	13.4	17	96
C3	48.2	14.5	12	85
C4	51.8	15.1	16	88
C5	57.2	14.8	25	96
C6	58.9	13.6	26	84

CEAP, clinical, aetiological, anatomical and pathophysiological

Most patients' daily activities (80.3%) involved standing for periods exceeding six hours. Varicose veins were identified in 52.1% of patients who remained standing for longer than six hours and in 46.3% of patients who stood for less than six hours. There was no significant difference between groups comparing pain and the number of hours standing. Oedema was present in 33.2% and 32.2% of individuals who remained standing for periods of more than and less than six hours per day, respectively.

A total of 25.3% of the participants regularly practiced some type of physical activity with the most common being: walking – 6.2%, gym – 18% and water aerobics – 7% (Table 3). Patients with CEAP C1 practiced physical activities most often and those classified as C5 or C6 the least.

**Table 3:** Physical activities practiced by the study participants

Exercise	Percentage (%)
Walking	46.2
Gym	18.0
Water aerobics	7.0
Football	4.8
Swimming	4.2
Running	3.2
Riding a bicycle	1.7
Pilates	1.9
Others	13

A higher percentage of varicose veins were found in individuals who did not exercise (49.4%) compared with those who regularly practiced physical activities (44.1%).

The main indications specialists cited for prescribing compression therapy were pain and discomfort of the leg in 56.2% of patients (Table 4). The main type of stocking indicated by physicians in 42.39% of cases was knee length.

**Table 4:** Indications used by specialists to prescribe elastic compression stockings

Indication	%	95% confidence interval
Pain and discomfort	56.2	54.6–57.9
Varicose veins	48.4	46.7–50.0
Oedema	34.1	32.5–35.7
After varicose vein surgery	22.9	21.5–24.3
Sclerotherapy of telangiectasias	17.5	16.3–18.8
Prevention of varicose veins	5.3	4.5–6.0
Prevention of deep venous thrombosis	2.3	1.8–2.9
Treatment of deep venous thrombosis	4.3	3.6–5.0
Prevention of varicose veins during pregnancy	1.6	1.2–2.0

The reduction in complaints of pain, discomfort, burning sensation and swelling of the legs while using elastic compression stockings (comparing first visit and return) was evaluated (Table 5). There was a good improvement in symptoms in more than 90% of cases.

**Table 5:** Improvement in signs and symptoms of pain, discomfort, burning sensation and swelling of the legs while using elastic compression stockings

Complaint	Good improvement (%)	Little improvement (%)	No improvement (%)
Pain	96.5	3.1	0.4
Discomfort	96.0	3.6	0.5
Burning	90.9	7.3	1.6
Oedema	95.1	3.7	1.1

The improvement in symptoms and clinical signs was higher for individuals who used compression stockings for periods exceeding six hours daily and for more than four weeks.

Adherence to treatment with the purchase of stockings was 89.3% of patients. Those who did not buy stockings claimed that the main reasons were the high price (6.8% cases), difficulty in finding the stockings (1.7%), dissatisfaction with the appearance of stockings (0.7%) and other reasons (1.5%).

Adherence to treatment over the four-week study period was 66.8% with 3.9% of patients adhering to treatment for less than one week. Daily use of the elastic compression stockings exceeded six hours for 59.4% of the patients. The main reason for stopping the use of stockings was the difficulty in putting them on (Table 6). Compression therapy was prescribed for just 30 days in some cases as in patients submitted to sclerosis of varicose veins (17.5%).

**Table 6:** Main reasons to stop using elastic compression stockings or to use them incorrectly

Reason	%	95% confidence interval
Difficulties to put the stockings on	71.3	68.7–73.9
Failure to remember	34.3	31.6–37.0
Hot legs	29.1	26.5–31.7
Dissatisfaction with the appearance	15.3	13.3–17.4
Worsening of the symptoms	1.3	0.6–1.9
Worsening of the oedema	1.2	0.6–1.8
Others	2.8	1.9–3.8

## Discussion

This study presents important data on the use of elastic stockings and adherence to treatment of private patients in different regions of Brazil. Thus these data are of fundamental importance to specialists when indicating compression stockings.

The first important fact is that the majority of patients (89.3%) purchased stockings showing that the treatment was accepted by most. Only 6.5% of patients said the stockings were too expensive while 1.4% had difficulty in finding them and 0.8% of patients did not buy because of the appearance. Data shows that stockings are well distributed in Brazil, are affordable to the majority of private patients and have an acceptable appearance for 99% at the time of purchase. More women use elastic compression stockings than men and around 85% of patients who were prescribed stockings had CEAP C3 or less.

Less than 70% of patients adhered to treatment during the entire four-week period with difficulties in putting the stockings on being a major reason to stop therapy. Additionally, the sensation of hot legs was the greatest discomfort in 29% and dissatisfaction with the appearance was important for 15.3% of patients. Moreover, worsening of symptoms and oedema occurred in 1.3% and in 1.2% of cases, respectively. These data reflect an important characteristic about the appearance; at the time of purchase it was not a problem but when using the garment the appearance troubled about 15% of patients. The goal of treatment was attained in 90% of patients and the symptoms worsened in only 2.5% of patients.

Another aspect to be considered is the strategies that physicians can use to encourage the regular use of stockings. It must be remembered that the elderly need help from a care-giver to put on the stockings which makes this a limiting factor of therapy.

It is also important to point out that this study was carried out in a tropical country and that the sensation of heat with the use of compression stockings discourages patients from continuing treatment. This fact should persuade manufacturers to look for other materials that are cooler in order to reduce the sensation of heat and thereby improve adherence to treatment.

Another point to be emphasized is that only 56.2% of this group of patients felt discomfort in the legs before using elastic stockings. The difficulties in putting them on, the appearance, feeling hot and the worsening of symptoms may have been the reason that 32.8% of patients did not adhere to treatment. Patients without symptoms may start having symptoms of discomfort and difficulties that lead to them stopping to use compression stockings. Thus, a more careful selection of patients may improve adherence.

Another important aspect discussed is related to the time that stockings should be used daily. About 60% of patients used stockings for more than six hours with better clinical outcomes. A study analysing the time of use of elastic stockings during the day showed that the use of compression therapy for a 12-hour period was more effective in reducing oedema than six hours of use or no use, and that the use of stockings for six hours was more effective in reducing oedema than not using at all.^8^ Oedema forms quicker early in the morning than later in the day^9^ and thus encouraging patients to use compression for longer periods is important as this may improve the results. The improving of the symptoms constituted an important factor for adhesion to treatment.

The severity of venous diseases of the patients in this study called our attention; 4% of patients, with a mean age between 45 and 50 years old, have or have had leg ulcers. At this clinical stage the involvement of changes in joint mobility is common and measures to improve mobility are suggested.^10^

About 18% of these patients already have skin lesions and require special care with prophylactic treatment so that the lesions do not evolve to venous leg ulcers. Hence, the use of elastic compression stockings is an important form of therapy. Again it is important to mention that this is a group of patients who are socioeconomically better off.

Regular physical activity helps maintain muscle tropism which is important in maintaining the efficiency of the venous pumps. In the current study, only 25% of patients practiced physical exercise with walking being the most common activity. Young people, in lower CEAP categories, seek to stay fit through exercise and activities. At older ages there is an increase in the CEAP and in even more advanced stages, physical limitations with leg ulcers and reduced joint mobility may restrain individuals from doing physical activities. The use of elastic stockings during walking should be encouraged as this increases venous working pressures between the skin and stocking.^11^

On analysing the results of this study, there was an association between increasing mean age and greater severity of CVI. This association has already been the subject of several published studies.^5,6,12^

In summary, adherence to treatment depends on several factors that include improvement in symptoms and a little discomfort related to the use of compression garments.

## Conclusions

Elastic compression stockings are available to the Brazilian population, have an acceptable appearance at the time of purchase and provide good results; however, some limitations regarding their use needs to be addressed.
